# Towards learning and memory risk assessment with human brain organoids: barriers and opportunities

**DOI:** 10.3389/ftox.2026.1783893

**Published:** 2026-04-10

**Authors:** Ronit Mohapatra, Dowlette-Mary Alam El Din, Hanyu Zhao, Thomas Hartung, Lena Smirnova

**Affiliations:** 1 Department of Environmental Health and Engineering, Bloomberg School of Public Health, Center for Alternatives to Animal Testing, Johns Hopkins University, Baltimore, MD, United States; 2 Department of Biochemistry and Molecular Biology, Bloomberg School of Public Health, Johns Hopkins University, Baltimore, MD, United States; 3 Center for Alternatives to Animal Testing-Europe, University of Konstanz, Konstanz, Germany

**Keywords:** heavy metals, learning, memory, brain organoids, neurotoxicity, new approach methodologies (NAMs), organoid intelligence

## Abstract

Neurodevelopmental conditions, including autism spectrum disorder, intellectual disability, and learning disabilities, as well as neurodegenerative disorders, affect millions of people in the United States alone. Both genetic and environmental factors contribute to their onset, yet traditional neurotoxicity testing often fails to identify specific risks or mechanisms underlying cognitive impairment. Human brain organoids (hBOs), also called neural organoids or brain microphysiological systems, are three-dimensional (3D) stem cell-derived models that recapitulate key features of human brain development and offer greater physiological relevance than traditional 2D *in vitro* or animal models. The emerging field of “organoid intelligence” integrates these systems with advanced bioengineering and artificial intelligence to model higher-order neural functions and assess learning and memory-relevant endpoints that were previously less explored *in vitro*. Despite this promise, we have identified four key barriers that hinder the application of hBOs for the hazard identification phase of functional neurotoxic risk assessments: [1] limited maturity and regional complexity, [2] lack of high-throughput defined procedures for assessing cognitive development and function *in vitro*, [3] limited standardization for reproducibility, and [4] challenges in translating *in vitro* results to human health outcomes. Here, we outline current efforts to overcome these challenges, i.e., scientific, technical, and regulatory advances. We also illustrate how hBO-based assays can be applied to advance both mechanistic understanding and the regulatory evaluation of environmental (developmental) neurotoxicants, using heavy metals as a model.

## 1 Introduction

Neurodevelopmental and neurodegenerative disorders impair cognitive functions, like learning, memory, or language capacity, and are increasing in prevalence (American Psychological Association, 2018; Li and Jönsson, 2025). In the United States, the prevalence of any diagnosed disability in children aged 3–17 years increased from 7.40% to 8.56% between 2019 and 2021 (Zablotsky et al., 2023). Globally, the age-adjusted prevalence of Alzheimer’s disease (AD) rose 148% between 1990 and 2019 (Li X. et al., 2022). Environmental exposures, particularly during sensitive developmental windows, are increasingly implicated (Smirnova et al., 2014; Short and Baram, 2019; Albores-Garcia et al., 2021; Trapecar et al., 2021; Dickerson et al., 2023; Xie et al., 2025), yet mechanisms remain difficult to resolve because human neural systems are challenging to model *in vitro.* Additionally, developmental trajectories diverge between humans and animal models, especially in regions supporting higher-order cognition, like the hippocampus (Davidson and Stevenson, 2024; White et al., 2024).

Current neurotoxicity evaluation still depends on animal assays that capture integrated systems-level behaviors (OECD, 2004; Vorhees and Williams, 2006; NAFTA et al., 2016; Bal-Price et al., 2018b). Learning and memory are among the most complex higher-order brain functions and remain difficult to model *in vitro*, whereas animal assays such as the Morris water maze, novel object recognition, and fear conditioning provide established proxies for specific cognitive domains (Lang et al., 2023; Vorhees and Williams, 2024). Continued refinement of controlled human-based *in vitro* systems that recapitulate key physiological features (such as electrochemical activity and synaptic plasticity) is nonetheless needed to enhance mechanistic interrogations of neurotoxicity while reducing reliance on animal studies and interspecies extrapolation (Serafini et al., 2024).

Therefore, the field is developing mechanistically anchored, human-relevant new approach methodologies (NAMs) (National Research Council (U.S.), 2007), including microphysiological systems (MPS) (Marx et al., 2016; 2020; 2025) and the OECD-supported developmental neurotoxicity *in vitro* battery (DNT IVB) (OECD, 2023). While *in vitro* human-based systems cannot yet recapitulate whole-organism cognitive behaviors, the molecular basis of brain plasticity, including neurogenesis, synaptic plasticity, neural network formation, adaptation, and dynamics, can be modeled and studied (Paşca et al., 2015; Leist et al., 2017; Yuan et al., 2023). However, current *in vitro* DNT assays provide limited coverage of network-level function and plasticity. This leaves a gap between hazard identification and cognition-related outcomes.

Human induced pluripotent stem cell (iPSC)-derived brain organoids (hBOs), a subset of MPS, recapitulate key physiological features of the developing brain (Lancaster et al., 2013; Camp et al., 2015; Boylin et al., 2024; Smirnova and Hartung, 2024). hBOs can recapitulate the molecular and cellular substrates of information processing, learning and memory, to identify and characterize neurological disease-relevant risks (Cai et al., 2023; Smirnova et al., 2023; Osaki et al., 2024; Alam El Din et al., 2025; van der Molen et al., 2025; Robbins et al., 2026). Emerging organoid intelligence (OI) paradigms extend this potential by coupling hBOs with advanced electro-optical interfaces and real-time analytics to examine how organoids process inputs and adapt over time (Smirnova et al., 2023; Alam El Din et al., 2024). Paired with modern AI-based methods, these platforms resolve complex activity patterns and detect changes in responsiveness, communication, and plasticity with high sensitivity. Proofs-of-concept already show reservoir-computing behavior in brain organoids and real-time, feedback-driven adaptation in neuronal networks (Cao, 2022; Cai et al., 2023; Robbins et al., 2026). Consequently, our group proposed integrating hBO-based plasticity assays into the DNT IVB (Alam El Din et al., 2024; Smirnova et al., 2024).

However, several improvements in scientific validity, scalability, reproducibility, and translation are still required before hBOs can be fully integrated into neurotoxicity risk assessment. Key limitations include: [1] limited maturation and regional complexity of hBOs, [2] lack of high-throughput defined procedures for assessing cognitive development and function *in vitro*, [3] insufficient standardization for reproducibility, and [4] difficulty translating *in vitro* results to human outcomes. Ethical, legal, and social considerations also influence adoption but fall outside the scope of this review (Hartung et al., 2023; Pichl et al., 2023). By outlining recent advances, remaining challenges, and practical applications, this review aims to guide development of hBO-based (developmental) neurotoxicity testing toward hazard and risk assessment within the broader MPS field.

## 2 Barriers in the application of human brain organoids for neurotoxicant testing and risk assessment

### 2.1 Limited maturity and regional complexity of human brain organoids

hBOs model human-specific molecular and cellular processes, including intercellular interactions and physiologically relevant composition, without interspecies differences (Mora-Bermúdez et al., 2016; Barreras et al., 2023; Saglam-Metiner et al., 2023). These systems have already been applied to evaluate neurotoxic exposures (e.g., Li et al., 2020; Modafferi et al., 2021; Choi et al., 2024; Huang et al., 2024), but they remain simplified models due to the absence of a full developmental niche and macro-anatomical organization. This limitation is underscored by transplantation studies in rats showing that host integration of cerebral organoids can accelerate maturation across multiple cell types beyond what is typically achieved *in vitro* (Revah et al., 2022). Most hNOs are cultured for weeks to over a year and still largely resemble fetal or early postnatal physiology (Lee et al., 2022), constraining applications to aging, neurodegeneration, or latent developmental effects (Urrestizala-Arenaza et al., 2024). Recently, hNOs have been cultured for up to 5 years, demonstrating a continuous increase in complexity and transcriptional ageing (Faravelli et al., 2025). Novel strategies to enhance hNO maturation rate include expanding glial populations (Sabate-Soler et al., 2022; Morales Pantoja et al., 2024) supplementing morphogenic factors, developing vasculature (Cakir et al., 2019; Shi et al., 2020) and incorporating microglia to generate immune-competent models (Rittenhouse et al., 2025; Table 1 therein). Complementary “neuro-aging proxies,” such as inducing age-associated neuroinflammation *via* monocyte perfusion in organoid-on-chip systems (Ao et al., 2022) may further improve relevance for later-life outcomes.

Cognition-relevant functions also depend on interregional specialization and connectivity (Qian et al., 2018; Magni et al., 2022; Susaimanickam et al., 2022; Kshirsagar et al., 2025). During embryogenesis, morphogen gradients (e.g., WNT, SHH, *etc.*) drive spatial patterning (Stiles and Jernigan, 2010; Nishimura and Li, 2024). Applying these same factors in guided differentiation protocols can generate region-specific organoids relevant to learning and memory, such as the cortex, hippocampus, thalamus, and striatum (Susaimanickam et al., 2022; Supplementary Table S1). These region-specific organoids can be fused into assembloids to model interregional circuitry, neuronal projections and subtype migration (Shellard and Mayor, 2019; Onesto et al., 2024; Paşca et al., 2024) (summarized in Table 1). For example, assembloid systems have been shown to form bidirectional thalamocortical and corticothalamic projections, resembling the reciprocal connectivity observed *in vivo* (Xiang et al., 2019; Onesto et al., 2024; Patton et al., 2024). However, fusion protocols introduce variability in the form of fusion efficiency and timing, which can shift developmental trajectories (Urrestizala-Arenaza et al., 2024; Lombardozzi et al., 2025). Alternatively, recent studies have demonstrated that organoids can form pharmacologically and electrically responsive neural networks *via* neural projections without direct fusion (“connectoids”) (Duenki and Ikeuchi, 2026).

**TABLE 1:** Efforts in developing region-specific human brain organoid systems related to learning and memory functions.

References	Brain region(s)	Model type	Developmental maturity (culture time)
Nityanandam et al. (2025)	Thalamus, cortex	Assembloid	Fetal (90 days)
Tran et al. (2025)	Striatum, midbrain	Assembloid	Fetal (60–90 days)
Walsh et al. (2025)	Cortex (ventral/dorsal forebrain)	Assembloid	Fetal (120 days)
McCrimmon et al. (2025)	Cortex, hippocampus, ganglionic eminence	Assembloid	Unspecified (120 days)
Sun et al. (2024)	Forebrain	Vascularized Assembloid	Late fetal/early postnatal (2.5 months)
Fitzgerald et al. (2024)	Cortex	Organoid	Fetal (6–10 months)
Wu et al. (2024a)	Striatum, midbrain	Assembloid	Embryonic (60–80 days)
Wu et al. (2024b)	Hippocampus	Organoid	Fetal, 22 gestational weeks (60–90 days)
Kiral et al. (2023)	Thalamus	Organoid	Fetal (112 days)
Eigenhuis et al. (2023)	Cortex	Organoid	Unspecified (up to 120 days)
Magni et al. (2022)	Cortex	Organoid	Mid fetal (3 months)
Chen et al. (2022)	Striatum, midbrain	Assembloid	Fetal (up to 110 days)
Gordon et al. (2021)	Cortex	Organoid	Postnatal (250–300 days)
Andersen et al. (2020)	Cortex, hindbrain	Assembloid	Unspecified (45–130 days)
Smits and Schwamborn (2020)	Midbrain	Organoid	Embryonic/fetal (up to 100 days)
Shi et al. (2020)	Cortex	Vascularized Organoid	Fetal (200 days)
Pomeshchik et al. (2020)	Hippocampus	Organoid	Unspecified (100 days)
Jacob et al. (2020)	Cortex, midbrain, hypothalamus, hippocampus, choroid plexus	Organoid	Embryonic/fetal (20–100 days)
Xiang et al. (2018)	Dorsal thalamus, cortex	Assembloid	Fetal (100 days)
Qian et al. (2018)	Forebrain, midbrain, hypothalamus	Organoid	Unspecified apart from “developmental” (300 days)
Renner et al. (2017)	Forebrain (dorsal/ventral)	Organoid	Embryonic/early fetal (33–229 days)
Xiang et al. (2017)	Medial ganglionic eminence, cortex	Assembloid	Unspecified (>18 days)
Monzel et al. (2017)	Midbrain	Organoid	Embryonic (61 days)
Watanabe et al. (2017)	Cortex, Basal ganglia	Organoid	Fetal, gestational week 14 (150 days)
Jo et al. (2016)	Midbrain	Organoid	Fetal, 2nd trimester (up to 130 days)
Camp et al. (2015)	Neocortex	Organoid	Fetal (12–13 weeks)
Sakaguchi et al. (2015)	Hippocampus	Organoid	Fetal (100 days)
Mariani et al. (2012)	Dorsal telencephalon	Organoid	Early embryonic (70 days)

Nonetheless, fully recapitulating the complexity of morphogenic gradients and full cellular diversity observed *in vivo* remains a challenge in guided differentiation (Onesto et al., 2024). Continued improvement in regional fidelity and maturation will benefit from controlled morphogen delivery through microfluidic gradients, automated maintenance (Voitiuk et al., 2025), and effective vascularization (Lee et al., 2018; Mansour et al., 2018) to support spatial patterning and physiological relevance.

### 2.2 Limited availability of defined procedures for assessing cognitive functions *in vitro*

Validation of *in vitro* test systems, including functional endpoints for hBOs (quantifiable measures of network dynamics and synaptic plasticity), requires that the system must recapitulate relevant human biology and that changes in molecular or cellular pathways leading to measurable endpoints must correspond to those observed in humans (Leist et al., 2012; Hartung et al., 2013; Bal-Price et al., 2018a; Piersma et al., 2018; Patterson et al., 2021; Blum et al., 2023). Both aspects require rigorous characterization, including assessments of reliability, inter-laboratory reproducibility, and ultimately predictivity, before a novel test system is considered validated. Based on this definition, no validated endpoints currently exist for hBO-based systems in functional neurotoxic risk assessments. Current efforts in this field are therefore best described as the development of defined procedures of functional assays. These efforts represent an essential precursor to future pre-validation and validation studies.

Synaptic plasticity is the cellular basis for learning and memory (Mansvelder et al., 2019) and thus is central to assessing risk to these functions. Currently, the only accepted *in vitro* functional DNT assay uses rat cortical neurons cultured on microelectrode arrays (MEAs) for longitudinal recording of neural network activity (Brown et al., 2016; Frank et al., 2017; Bal-Price et al., 2018a; Bal-Price et al., 2018b). This assay quantifies toxicant-induced changes in bursting and spiking but is constrained by its non-human origin and has not yet been used to assess synaptic plasticity. For human-based functional endpoints, Bartmann et al. developed a similar MEA-based neural network formation assay using hiPSC-derived neurons (Bartmann, 2023). To date, this has not been extended to hBOs nor adapted to include plasticity assessments.

Beyond regulatory testing contexts, several proof-of-concept studies have demonstrated adaptation-like behaviors in human neuronal systems. Kagan et al. reported plasticity-driven response adjustment in a 2D culture of hiPSC-derived neurons (Kagan et al., 2022). More recently, early progress towards human brain organ-on-chip platforms for safety testing related to neurodegeneration has been reported (Autar et al., 2025). Similar efforts have extended to organoids in basic science research. For example, Robbins et al. demonstrated closed-loop activity modulation (as a proxy for goal-directed learning) in cortical organoids (Robbins et al., 2026), Cai et al., showed organoids can act as a reservoir for computing and information processing (Cai et al., 2023), and Rountree et al., showed organoids adapted their responses based on electrical stimulation (Rountree et al., 2025).

Consistent with these findings, hBOs have been repeatedly shown to exhibit spontaneous electrical activity and network formation that can be modulated chemically or electrically (Trujillo et al., 2019; Sharf et al., 2022; Osaki et al., 2024; Alam El Din et al., 2025; Rountree et al., 2025; van der Molen et al., 2025). Despite this progress, there are still no defined procedures for this context of use: assessing cognitive functions like learning and memory *in vitro* for regulatory purposes.

To address this, we previously proposed an OI framework for developing hBO-based functional endpoints to quantify synaptic plasticity following toxicant exposure (Smirnova et al., 2023; Alam El Din et al., 2024). These endpoints incorporate open- and closed-loop experiments integrating hBOs with MEA devices (Smirnova et al., 2023; Alam El Din et al., 2024). In open-loop experiments, hNOs are stimulated and responses recorded. In closed-loop configurations, stimulation is adjusted in real time based on recorded activity (Tessadori et al., 2012). Together, validation of these approaches will enable quantification of toxicant-induced alterations in information-processing mechanisms.

Early progress towards the development of defined procedures for assessing cognitive development and function *in vitro* has been made by demonstrating learning- and memory-related cellular processes in hBOs (Zafeiriou et al., 2020; Osaki et al., 2024; Patton et al., 2024; Alam El Din et al., 2025; Rountree et al., 2025), fulfilling the first criterion of biological relevance. Additional work to meet the second criterion will involve applying these endpoints to toxicant screening and disease models. Screening is currently constrained by tradeoffs among electrophysiology platforms, including patch clamp, microelectrode array (MEA) devices, and calcium (Ca^2+^) imaging (Paşca et al., 2015; Li et al., 2017; Sakaguchi et al., 2019; Osaki et al., 2024). Patch clamp provides high-resolution measurements but is not scalable for this use case. Longitudinal, network-level recordings with electrical stimulation in MEAs are currently low-to-medium throughput, whereas Ca^2+^ imaging can scale to high-throughput screening but offers limited capacity for patterned electrical stimulation needed to probe synaptic plasticity mechanisms. Advances will require MEA technologies that combine high-density (HD) recording with increased well capacity. New 3D-MEAs and integrated mesh electrodes also increase global resolution of hBO recordings, though they remain low throughput (Soscia et al., 2020; Passaro and Stice, 2021; Huang et al., 2022; Li T. L. et al., 2022).

### 2.3 Limited standardization and reproducibility in the generation and analysis of brain organoids

Protocols for generating hBOs vary widely across laboratories and are often inconsistent in cellular composition, morphology, relative maturity, and stress responses (Quadrato et al., 2017; Bhaduri et al., 2020). hNOs exhibit stochastic self-organization and cell line-specific differences that can obscure subtle toxicant effects without rigorous experimental control (Velasco et al., 2019; Yoon et al., 2019). Activation of endoplasmic reticulum stress pathways *in vitro* has been shown to alter hBO cell-type specification, which may exacerbate variability (Bhaduri et al., 2020). However, transcriptomic analysis has demonstrated that certain hBO protocols can generate reproducible, brain-like cellular compositions (Velasco et al., 2019). Guided differentiation approaches (Table 1) may further enhance reproducibility by using controlled chemical patterning rather than spontaneous differentiation, particularly when protocol complexity is minimized (Eigenhuis et al., 2023; Fitzgerald et al., 2024). Additionally, Ramani et al. recently developed a method to rapidly produce thousands of comparable organoids per batch while minimizing stress pathway activation (Ramani et al., 2024).

Quality assurance frameworks for MPS, including hBOs, are emerging based on established *in vitro* standards (Ekert et al., 2020; Pamies et al., 2022; 2024). Translating these principles into hBO-specific performance standards, such as minimal criteria for cell-type composition and response to positive control neurotoxicants (Mundy and Crofton, 2024), would improve reproducibility and generate sufficient reference data for evaluation (Harrill et al., 2024). Current efforts to standardize *in vitro* reporting (Antes, 2010; OECD, 2018; Percie du Sert et al., 2020; The RIVER working Group, 2023; Mohapatra et al., 2025) will be essential to build confidence, support validation, and enable regulatory integration (Leist et al., 2012; Harrill et al., 2024).

Standardization challenges also extend downstream, particularly in electrophysiology. MEA platforms often rely on proprietary or lab-specific spike-sorting algorithms. Harmonization of the data processing pipeline and statistical analysis would increase reproducibility and consistency across labs (Sukiban et al., 2019). AI algorithms are likely to improve reproducibility, throughput, and accuracy in these analyses (Smirnova et al., 2023; Maramraju et al., 2024; Sewell et al., 2024; Wang et al., 2025).

### 2.4 Challenging translation of *in vitro* results to adversity in human health

For risk assessment, extrapolating *in vitro* findings to human-relevant outcomes remains a challenge for adopting MPS technology, particularly hBOs (NC3Rs, 2025). Certain neurological disorder phenotypes, such as atypical socialization in ASD, cannot be modeled *in vitro*, but hBOs nonetheless offer a promising path forward. Benchmarking involves assessing how well neurotoxicity outcomes from experimental models predict human clinical data or historical animal-study findings, for example, comparison to human cell atlases at various stages of development (Steger-Hartmann and Raschke, 2020; Childs et al., 2022; Park et al., 2022). As hBO datasets grow, benchmarks will strengthen and support more informative experimental design and improve translation to human health.

Epigenomics and other “omics”-based technologies have been used for biomarker identification, as early-life exposures can leave persistent epigenetic signatures associated with later cognitive deficits or neurodegeneration (Baccarelli et al., 2023; Zhang et al., 2025). For example, high-resolution transcriptomic (Mathys et al., 2019; Murdock and Tsai, 2023) and epigenetic (Smith et al., 2021) profiles associated with AD have demonstrated the feasibility of linking molecular signatures with disease trajectories. Identifying parallel biomarkers across hBOs and epidemiological datasets could meaningfully strengthen confidence in hBO-based predictions of long-term neurodevelopmental and neurodegenerative risks (Kang et al., 2024).

Linking *in vitro* exposures in hBOs to human-relevant doses is also critical for risk assessment (Roberts et al., 2022). Physiologically based pharmacokinetic (PBPK) models, which simulate chemical absorption and distribution across organs, help bridge this gap (Sager et al., 2015; Yang et al., 2022). PBPK models integrated with *in vitro* and *in silico* blood-brain barrier (BBB) systems have already been applied to estimate dosage in neurotoxicity studies (Nzou et al., 2018; Illa et al., 2025). Achieving regulatory-grade use will require standardized PBPK models, which consider confounding factors (age, sex, genotype, *etc.*) to integrate with hBOs (Sung et al., 2010; Cirit and Stokes, 2018). Even with appropriate exposure schemes, hBO-based assessments must also incorporate quantitative *in vitro-in vivo* extrapolation to connect organoid functional readouts to adverse outcome pathways (AOPs) and to population-level cognitive outcomes reported in epidemiological studies (Chang et al., 2022; Cattelani et al., 2024).

Regulators continue to rely primarily on animal and epidemiological studies to identify cognitive risks (NAFTA et al., 2016; OECD, 2023). As regulatory uptake of DNT NAMs increasingly relies on case-study-based workflows that integrate assay batteries, exposure modeling, and AOP reasoning (Tal et al., 2024; Zwartsen et al., 2025), hBO assays should be incorporated into these frameworks rather than treated as stand-alone tools for DNT and cognitive risk assessments. As in animal studies, where guidelines such as ICH M3(R2) require two species to ensure the reliability of non-clinical results (FDA, 2009; Van Norman, 2019), hBO-based risk assessment may require complementary NAMs to increase the weight of evidence for neurotoxicity.

## 3 Discussion: applications of human brain organoids in heavy metal neurotoxicity

Heavy metals such as lead (Pb), methylmercury (MeHg), and manganese (Mn) are among the best-characterized neurotoxicants, with evidence linking early-life exposure to lower IQ, poorer executive function, altered behavior, and neurodegeneration, even at levels previously considered safe (Grandjean and Landrigan, 2014; Bellinger, 2018; Oliveira et al., 2018; Shen et al., 2021; Heng et al., 2022; da Silva et al., 2025). Certain metals, like cadmium (Cd) and hexavalent chromium (Cr(VI)), are high-priority hazards associated with cognitive delays (Heng et al., 2022; Jia et al., 2024), but much less mechanistically characterized in neurological systems (Wise et al., 2022). The neurotoxic mechanisms of various heavy metals have been reviewed extensively (Balali-Mood et al., 2021; Singh and Sharma, 2021; Virgolini and Aschner, 2021; Panzenhagen et al., 2024; Song et al., 2025). They provide practical “anchor chemicals” for illustrating where hBOs could contribute to neurotoxic risk assessment. Here, we focus on a subset of metals, two well-studied (Pb, MeHg) and two less well-characterized (Cd, Cr), as representative cases for advancing hBO-based neurotoxicity assessments.

Across metals, shared neurotoxic effects (e.g., oxidative stress, mitochondrial dysfunction, excitatory–inhibitory imbalance, and Ca^2+^ signaling disruption) coexist with metal-specific action, such as Pb-induced antagonism of NMDA receptor 2A (Nihei and Guilarte, 1999; Moyano et al., 2018). However, key uncertainties persist about dominant mechanisms under mixed exposures, the causality of epigenetic changes, and how early molecular disruptions lead to latent cognitive deficits. Functional readouts from human-relevant *in vitro* models are needed to connect physiological perturbations to downstream outcomes, including altered neuroplasticity and network formation, under tightly controlled conditions. We next highlight how advances in hBO technology can enhance the evidence base for public health action regarding heavy metal risks.

Proposed mechanisms for Pb and MeHg-induced neurotoxicity include persistent epigenomic alterations (Wang et al., 2020; Go et al., 2021; Wei et al., 2024), oxidative stress-driven developmental errors (Abubakar et al., 2019; Fujimura et al., 2020; Nishimura et al., 2021), and neurotransmitter imbalance during neurodevelopment (Chen et al., 2021; Yadav et al., 2022; Wang et al., 2023). Region-specific hBO/assembloid models with accelerated maturation could confirm these hypotheses in developmentally relevant circuits and probe compartmentalized toxicity shaped by exposure route (e.g., olfactory uptake vs. BBB transport) and receptor expression (Branca et al., 2019; Corbo et al., 2025). This potential application is consistent with region-specific phenotypes observed in assembloid models of multifactorial disorders (Mitchell et al., 2024; McCrimmon et al., 2025).

As hBOs can model cellular aspects of cognitive function, they can potentially reveal how heavy-metal-induced disruptions in synaptic plasticity (LTP/LTD) alter network activity in open-loop assays, while closed-loop reinforcement-like paradigms provide *in vitro* proxies of learning and memory. Consequently, developing robust open- and closed-loop electrophysiological assays will be critical for identifying AOPs linked to cognitive impairment. Additionally, hBOs could model gene-environment interactions and help pinpoint key developmental windows of exposure (Kumar, 2025). The toxicology of metal mixtures is also insufficiently characterized, and increasing the throughput of hBO-based assays may enable systematic investigation of combined metal effects on brain development and function (Rai et al., 2010; Karri et al., 2016; Chandra et al., 2026).

**FIGURE 1:**
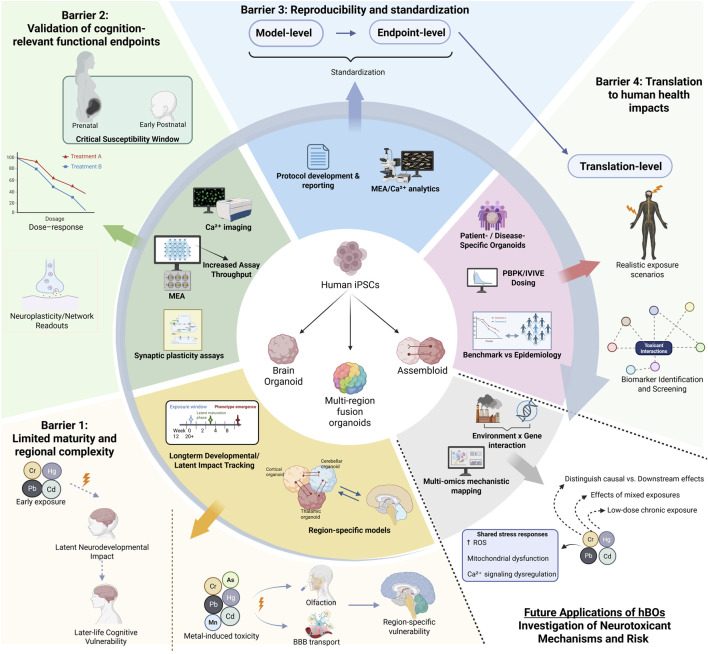
Barriers and opportunities for the application of human brain organoids in cognitive risk assessment and heavy metal toxicology. Schematic overview of the four major barriers identified in this review: [1] limited maturation and regional complexity of hBOs, [2] lack of high-throughput defined procedures for assessing cognitive development and function *in vitro*, [3] insufficient standardization for reproducibility, and [4] difficulty translating *in vitro* results to human outcomes. Elements within the central circle represent broadly applicable technological and methodological advances to improve human brain organoid (hBO) performance, whereas elements outside the circle illustrate specific applications of hBOs in heavy metal neurotoxic risk assessment. Abbreviations: Hg, mercury; Pb, lead; As, arsenic; Mn, manganese; Cd, cadmium; Cr, chromium; iPSC, induced pluripotent stem cell; MEA, microelectrode array; Ca^2+^, calcium ion; PBPK, Physiology-based pharmacokinetic modeling; IVIVE, *in vitro-*to*-in vivo* extrapolation; ROS, reactive oxygen species. Created in BioRender. Mohapatra, R and Zhao, H (2026) https://BioRender.com/ggfbia7.

To ensure comparability and translational value, standardized analysis and reporting must advance in parallel. Increasing model diversity introduces variability but may also enhance versatility, revealing toxicant-specific vulnerabilities. For example, hBOs with BBB-like features can model Cd-induced barrier disruption during development (Chandravanshi et al., 2021). Cross-study interpretation depends on alignment with compiled hBO datasets, including single-cell and transcriptomic resources such as the Human Neural Organoid Cell Atlas (He et al., 2024), which may also support the identification of population-level biomarkers of metal-induced neurotoxicity (Childs et al., 2022). Patient-derived organoids will potentially enable further investigation of individual variation in environmental responses, advancing precision toxicology and personalized risk assessment (Baccarelli et al., 2023; Strand et al., 2024). These potential applications and remaining barriers are summarized in Figure 1.

## 4 Conclusion

Modern hBOs provide an advanced, human-relevant platform for studying brain development and function and offer a promising route to integrate functional neurobiology into existing risk-assessment frameworks. While modeling whole-organism cognitive behaviors remains aspirational, emerging hBO-based functional assays can already link physiological disruption to functional deficits in learning- and memory-related endpoints like network connectivity/dynamics and synaptic plasticity. This supports hazard identification and mechanistic interpretation of neurotoxicants (including heavy metals). Broader adoption is limited by incomplete model complexity, a shortage of scalable cognition-relevant functional assays, and insufficient standardization for translation to human health. Building on advances in maturation, throughput, and harmonized performance criteria (Supplementary Table S2) will help close these gaps.
