# Supplementary material for: Towards learning and memory risk assessment with human brain organoids: barriers and opportunities
Source: Front Toxicol. 2026 Apr 10;8:1783893. doi: 10.3389/ftox.2026.1783893 (PMC13105461; doi:10.3389/ftox.2026.1783893)
Supplement: Supplementary file 1 [file Supplementaryfile1.docx]

Supplementary Material

# Supplementary Data

This is the supplemental material for the mini-review “Towards Human Brain Organoids for Learning and Memory Risk Assessment: Barriers and Opportunities” It includes supplemental information on human brain anatomy and function which was not included in the main review (Table S1) and a summarization of actionable conclusions from the main text (Table S2)

# Supplementary Figures and Tables

## Supplementary Tables

**Supplementary Table S1. Anatomical and Functional Relevance of Brain Regions Targeted in Table 1 of Main Text**

| Brain Region | Function Relating to Learning and Memory | Relevant References |
| --- | --- | --- |
| Thalamus | - Integration and trafficking of information between brain regions via reciprocal connections (notably with the cortex) - Thalamic volume and connectivity directly correlate with information processing and overall cognitive performance | (Fama and Sullivan, 2015; Saalmann and Kastner, 2015; Wang et al., 2025) |
| Cortex/Neocortex | - The neocortex is the largest part of the cerebral cortex in humans - Interpretation of sensory inputs - Generation of abstract thought, language, and reasoning - Facilitation of memory and emotional connections | (Staiger, 2015; Brodal, 2016; Galakhova et al., 2022) |
| Striatum | - Critical dopaminergic signaling for reinforcement learning, habit formation and working memory | (Scimeca and Badre, 2012; Burton et al., 2015) |
| Midbrain | - Processing of audio and visual data and connection with cortex for further processing - Dopaminergic signaling to encode reinforcement learning and motivations - Overlapping function with hippocampus for memory encoding and integration - Serotonergic and cholinergic systems to encode alertness | (Shohamy and Wagner, 2008; D’Ardenne et al., 2012; Walter and Shaikh, 2014) |
| Hippocampus | - Necessary structure for memory formation and consolidation - Generation of episodic memories and major contributor to spatial navigation capacity - Integration into the reward system for goal-directed behavior and relational memory - Responsible for pattern separation and completion as well as temporal organization - Central region in brain studied for long-term plasticity | (Bird and Burgess, 2008; Anand and Dhikav, 2012; Pierrefiche, 2019) |
| Forebrain (ventral/dorsal) | - Extremely broad classification of brain region which includes: cerebral cortex and limbic system (dorsal); olfactory bulb, basal ganglia, septum, hypothalamus and prethalamus (ventral) | (Khwaja et al., 2011) |
| Basal Ganglia | - Larger structure of primarily dopaminergic neural networks containing the striatum | (Packard and Knowlton, 2002) |
| Hindbrain | - Contains cerebellum which coordinates motor learning and reflexive memory (“muscle memory”) | (Saab, 2009; Rinaman, 2011) |
| Hypothalamus | - Hormonal modulation of synaptic strength and memory encoding - Integration of behavior and behavioral signals toward reward cues (e.g. in fear conditioning) | (Kosse and Burdakov, 2019; Burdakov and Peleg-Raibstein, 2020; Sharpe, 2024) |
| Choroid Plexus | - Regulation of brain microenvironment and cerebrospinal fluid production and regulation | (Gong et al., 2025) |
| Dorsal Telencephalon | - Developmental precursor to the cerebral cortex (neocortex), hippocampus, choroid plexus and amygdala | (Hébert and Fishell, 2008; Cavodeassi et al., 2009) |

**Supplemental Table S2. Actionable Conclusions for Human Brain Organoid Development for Neurotoxicity Applications Summarized from Main Text**

| **Barrier** | **Actionable Conclusions** |
| --- | --- |
| **Limited maturity and regional complexity** | - Accelerate maturation using defined engineered/biochemical additions   - Evaluate maturity against explicit *in vivo* benchmarks - Increase regional fidelity and circuit relevance via controlled patterning which prioritizes morphogen gradient control using engineered microfluidic systems - Model interregional connectivity with circuit‑competent constructs (assembloids or connectoids) - Scale and stabilize long cultures through automation (maintenance/monitoring) to support maturation and reproducibility as models extend to longer timeframes |
| **Limited availability of defined procedures to assess cognition relevant function *in vitro*** | - Prioritize “defined procedures” (DPs) before validation. Emphasizing biological relevance, reliability and inter-lab reproducibility/predictivity before claiming fully validated assays to assess cognition-related endpoints in hBOs - Implementation of open- and closed-loop stimulation/recording of hBOs to quantify toxicant effects on information processing and plasticity - Match electrophysiological platform choice to context of use.   - Use MEAs where stimulation and longitudinal network readouts are required, use Ca^2+^ imaging where high-throughput/high-content screening is the priority, use patch-clamp for specific confirmations of effects at high resolution rather than as a screening tool   - Invest in next generation electrophysiology to close throughput gaps, especially HD-MEA + higher well capacity and emerging 3D/mesh interfaces |
| **Limited standardization and reproducibility** | - Define and implement hBO-specific quality assurance/control performance standards - Adopt fit-for-purpose quality management and reporting frameworks from MPS and cell‑culture standards to support reproducibility claims and establish traceable experimental control across batches/labs - Harmonize electrophysiology data processing pipelines, including spike sorting and statistical approaches, to avoid “platform-specific” conclusions driven by proprietary or lab-specific algorithms   - Potential to incorporate AI/machine learning approaches for standardization (not just discovery) of data analysis |
| **Challenging translation of *in vitro* results to human outcomes** | - Benchmark hBO outputs to human reference datasets to quantify relevance for a given context of use/application of hBOs - Develop cross-system biomarkers and signatures relying on omics databases compared across hBOs and human datasets to strengthen confidence in long-term predictions of latent effects *in vitro* - Use exposure modeling and IVIVE to align hBO dosing and timing with real‑world human exposures for more interpretable risk‑assessment results. - Validate hBOs with individual case-study experiments for known neurotoxicants - Embed hBO assays in integrated NAM workflows, rather than positioning them as standalone tests, to build weight of evidence |

## Supplementary References

Anand, K. S., and Dhikav, V. (2012). Hippocampus in health and disease: An overview. *Ann. Indian Acad. Neurol.* 15, 239. doi: 10.4103/0972-2327.104323

Bird, C. M., and Burgess, N. (2008). The hippocampus and memory: insights from spatial processing. *Nat. Rev. Neurosci.* 9, 182–194. doi: 10.1038/nrn2335

Brodal, P. (2016). “Functions of the Neocortex,” in *The Central Nervous System*, ed. P. Brodal (Oxford University Press), 0. doi: 10.1093/med/9780190228958.003.0034

Burdakov, D., and Peleg-Raibstein, D. (2020). The hypothalamus as a primary coordinator of memory updating. *Physiol. Behav.* 223, 112988. doi: 10.1016/j.physbeh.2020.112988

Burton, A. C., Nakamura, K., and Roesch, M. R. (2015). From ventral-medial to dorsal-lateral striatum: Neural correlates of reward-guided decision-making. *Neurobiol. Learn. Mem.* 117, 51–59. doi: 10.1016/j.nlm.2014.05.003

Cavodeassi, F., Kapsimali, M., Wilson, S. W., and Young, R. M. (2009). “Forebrain: Early Development,” in *Encyclopedia of Neuroscience*, (Elsevier), 321–325. doi: 10.1016/B978-008045046-9.01065-2

D’Ardenne, K., Eshel, N., Luka, J., Lenartowicz, A., Nystrom, L. E., and Cohen, J. D. (2012). Role of prefrontal cortex and the midbrain dopamine system in working memory updating. *Proc. Natl. Acad. Sci.* 109, 19900–19909. doi: 10.1073/pnas.1116727109

Fama, R., and Sullivan, E. V. (2015). Thalamic structures and associated cognitive functions: Relations with age and aging. *Neurosci. Biobehav. Rev.* 54, 29–37. doi: 10.1016/j.neubiorev.2015.03.008

Galakhova, A., Hunt, S., Wilbers, R., Heyer, D., de Kock, C., Mansvelder, H., et al. (2022). Evolution of cortical neurons supporting human cognition. *Trends Cogn. Sci.* 26, 909–922. doi: 10.1016/j.tics.2022.08.012

Gong, Z., Bilgel, M., Faulkner, M. E., Bae, J., Laporte, J. P., Guo, A., et al. (2025). Associations Between Choroid Plexus Integrity and Cognitive Decline in Aging: Insights from Advanced MRI Analysis. *Alzheimers Dement.* 20, e093777. doi: 10.1002/alz.093777

Hébert, J. M., and Fishell, G. (2008). The genetics of early telencephalon patterning: some assembly required. *Nat. Rev. Neurosci.* 9, 678–685. doi: 10.1038/nrn2463

Khwaja, O. S., Pomeroy, S. L., and Ullrich, N. J. (2011). “Development of the Nervous System,” in *Fetal and Neonatal Physiology*, (Elsevier), 1745–1763. doi: 10.1016/B978-1-4160-3479-7.10160-0

Kosse, C., and Burdakov, D. (2019). Natural hypothalamic circuit dynamics underlying object memorization. *Nat. Commun.* 10, 2505. doi: 10.1038/s41467-019-10484-7

Packard, M. G., and Knowlton, B. J. (2002). Learning and memory functions of the Basal Ganglia. *Annu. Rev. Neurosci.* 25, 563–593. doi: 10.1146/annurev.neuro.25.112701.142937

Pierrefiche, O. (2019). “Synaptic Plasticity in the Hippocampus and Alcohol Exposure During Brain Development,” in *Neuroscience of Alcohol*, (Elsevier), 99–108. doi: 10.1016/B978-0-12-813125-1.00011-8

Rinaman, L. (2011). Hindbrain noradrenergic A2 neurons: diverse roles in autonomic, endocrine, cognitive, and behavioral functions. *Am. J. Physiol.-Regul. Integr. Comp. Physiol.* 300, R222–R235. doi: 10.1152/ajpregu.00556.2010

Saab, C. Y. (2009). *The Hindbrain*. Infobase Publishing.

Saalmann, Y. B., and Kastner, S. (2015). The cognitive thalamus. *Front. Syst. Neurosci.* 9. doi: 10.3389/fnsys.2015.00039

Scimeca, J. M., and Badre, D. (2012). Striatal Contributions to Declarative Memory Retrieval. *Neuron* 75, 380–392. doi: 10.1016/j.neuron.2012.07.014

Sharpe, M. J. (2024). The Cognitive (lateral) Hypothalamus. *Trends Cogn. Sci.* 28, 18–29. doi: 10.1016/j.tics.2023.08.019

Shohamy, D., and Wagner, A. D. (2008). Integrating Memories in the Human Brain: Hippocampal–Midbrain Encoding of Overlapping Events. *Neuron* 60, 378–389. doi: 10.1016/j.neuron.2008.09.023

Staiger, J. F. (2015). Cortical GABAergic Neurons. *Neurosci. Biobehav. Psychol.* 2, 69–80. doi: 10.1016/B978-0-12-397025-1.00202-5

Walter, B. L., and Shaikh, A. G. (2014). Midbrain. *Encycl. Neurol. Sci.*, 28–33. doi: 10.1016/B978-0-12-385157-4.01161-1

Wang, B. A., Li, S., Halassa, M. M., and Pleger, B. (2025). The unique role of the associative thalamus in cognitive processing. *Brain Res. Bull.* 229, 111432. doi: 10.1016/j.brainresbull.2025.111432
